# Fidaxomicin jams *Mycobacterium tuberculosis* RNA polymerase motions needed for initiation via RbpA contacts

**DOI:** 10.7554/eLife.34823

**Published:** 2018-02-26

**Authors:** Hande Boyaci, James Chen, Mirjana Lilic, Margaret Palka, Rachel Anne Mooney, Robert Landick, Seth A Darst, Elizabeth A Campbell

**Affiliations:** 1 The Rockefeller UniversityNew YorkUnited States; 2 Department of BiochemistryUniversity of Wisconsin-MadisonMadisonUnited States; 3 Department of BacteriologyUniversity of Wisconsin-MadisonMadisonUnited States; National Institute of Child Health and Human DevelopmentUnited States

**Keywords:** cryo-electron microscopy, Fidaxomicin, Mycobacterium tuberculosis, RbpA, RNA polymerase, Other

## Abstract

Fidaxomicin (Fdx) is an antimicrobial RNA polymerase (RNAP) inhibitor highly effective against *Mycobacterium tuberculosis* RNAP in vitro, but clinical use of Fdx is limited to treating *Clostridium difficile* intestinal infections due to poor absorption. To identify the structural determinants of Fdx binding to RNAP, we determined the 3.4 Å cryo-electron microscopy structure of a complete *M. tuberculosis* RNAP holoenzyme in complex with Fdx. We find that the actinobacteria general transcription factor RbpA contacts fidaxomycin, explaining its strong effect on *M. tuberculosis*. Additional structures define conformational states of *M. tuberculosis* RNAP between the free apo-holoenzyme and the promoter-engaged open complex ready for transcription. The results establish that Fdx acts like a doorstop to jam the enzyme in an open state, preventing the motions necessary to secure promoter DNA in the active site. Our results provide a structural platform to guide development of anti-tuberculosis antimicrobials based on the Fdx binding pocket.

## eLife digest

Tuberculosis (TB) is an infectious disease that affects over ten million people every year. The *Mycobacterium tuberculosis* bacteria that cause the disease spread through the air from one person to another and mainly infect the lungs. Although curable, TB is difficult to eradicate because it is remarkably widespread, with one third of the world’s population estimated to carry the bacteria.

Treatment for TB involves a mix of antibiotics that should be taken for several months to a year. The number of multidrug-resistant TB cases, where the infection is not treatable by the common cocktail of antibiotics, is rapidly increasing. There is therefore a need to discover new drugs that can kill the *M. tuberculosis* bacteria.

An antibiotic called fidaxomicin is used to treat intestinal infections. Although it can kill *Mycobacterium tuberculosis* cells in culture, it is not absorbed from the intestines to the blood and thus cannot reach the lungs to kill the bacteria. It may be possible to change the structure of the drug so that it can enter the bloodstream. Before this can be done, researchers need to understand exactly how fidaxomicin kills the bacteria so that they know which parts of the drug they can alter without making it less effective.

Fidaxomicin kills bacterial cells by binding to an enzyme called RNA polymerase. The antibiotic prevents the enzyme from reading and ‘transcribing’ DNA to form molecules that are essential for life. To learn more about how fidaxomicin has this effect, Boyaci, Chen et al. used cryo-electron microscopy to look at structures of the *M. tuberculosis* RNA polymerase in different states, including when it was bound to fidaxomicin.

The structures reveal the chemical details of the interactions between the RNA polymerase and the antibiotic. The two molecules bind to each other through a region of the RNA polymerase that is unique to *M. tuberculosis* and closely related bacteria. Fidaxomicin acts like a doorstop to jam the RNA polymerase in an open state that cannot bind to DNA and transcribe genes.

Medicinal chemists could now build on these findings to develop new drugs that might treat TB, either by modifying fidaxomicin or designing new antibiotics that bind to the same region of the RNA polymerase. Because the fidaxomicin-binding region of the RNA polymerase is specific to *M. tuberculosis* new antibiotics could be tailored towards the bacteria that have a minimal effect on a patient’s normal gut bacteria.

## Introduction

The bacterial RNA polymerase (RNAP) is a proven target for antibiotics. The rifamycin (Rif) class of antibiotics, which inhibit RNAP function, is a lynchpin of modern tuberculosis (TB) treatment (Chakraborty and Rhee, 2015). TB, caused by the infectious agent *Mycobacterium tuberculosis* (*Mtb*), is responsible for almost 2 million deaths a year. It is estimated that one third of the world is infected. Mortality from TB is increasing, partly due to the emergence of strains resistant to Rifs (Rif^R^) (Zumla et al., 2015). Hence, additional antibiotics against Rif^R^ *Mtb* are needed.

Fidaxomicin (Fdx; also known as Dificimicin, lipiarmycin, OPT-80, PAR-101, or tiacumicin), an antimicrobial in clinical use against *Clostridium difficile* (*Cdf*) infection (Venugopal and Johnson, 2012), functions by inhibiting the bacterial RNAP (Talpaert et al., 1975). Fdx targets the RNAP 'switch region', a determinant for RNAP inhibition that is distinct from the Rif binding pocket (Srivastava et al., 2011), and Fdx does not exhibit cross-resistance with Rif (Gualtieri et al., 2009, 2006; Kurabachew et al., 2008; O'Neill et al., 2000). The switch region sits at the base of the mobile RNAP clamp domain and, like a hinge, controls motions of the clamp crucial for DNA loading into the RNAP active-site cleft and maintaining the melted DNA in the channel (Chakraborty et al., 2012; Feklistov et al., 2017). Fdx is a narrow spectrum antibiotic that inhibits Gram-positive anaerobes and mycobacteria (including *Mtb*) much more potently than Gram-negative bacteria (Kurabachew et al., 2008; Srivastava et al., 2011), but the clinical use of Fdx is limited to intestinal infections due to poor bioavailability (Venugopal and Johnson, 2012). Addressing this limitation requires understanding the structural and mechanistic basis for Fdx inhibition, which is heretofore unknown. Here, we used single-particle cryo-electron microscopy (cryo-EM) to determine structures of *Mtb* transcription initiation complexes in three distinct conformational states, including a complex with Fdx at an overall resolution of 3.4 Å. The results define the molecular interactions of *Mtb* RNAP with Fdx as well as the mechanistic basis of inhibition, and establish that RbpA, an Actinobacteria-specific general transcription factor (GTF), is crucial to the sensitivity of *Mtb* to Fdx.

## Results

### Fdx potently inhibits mycobacterial TICs in vitro

**Figure 1.**
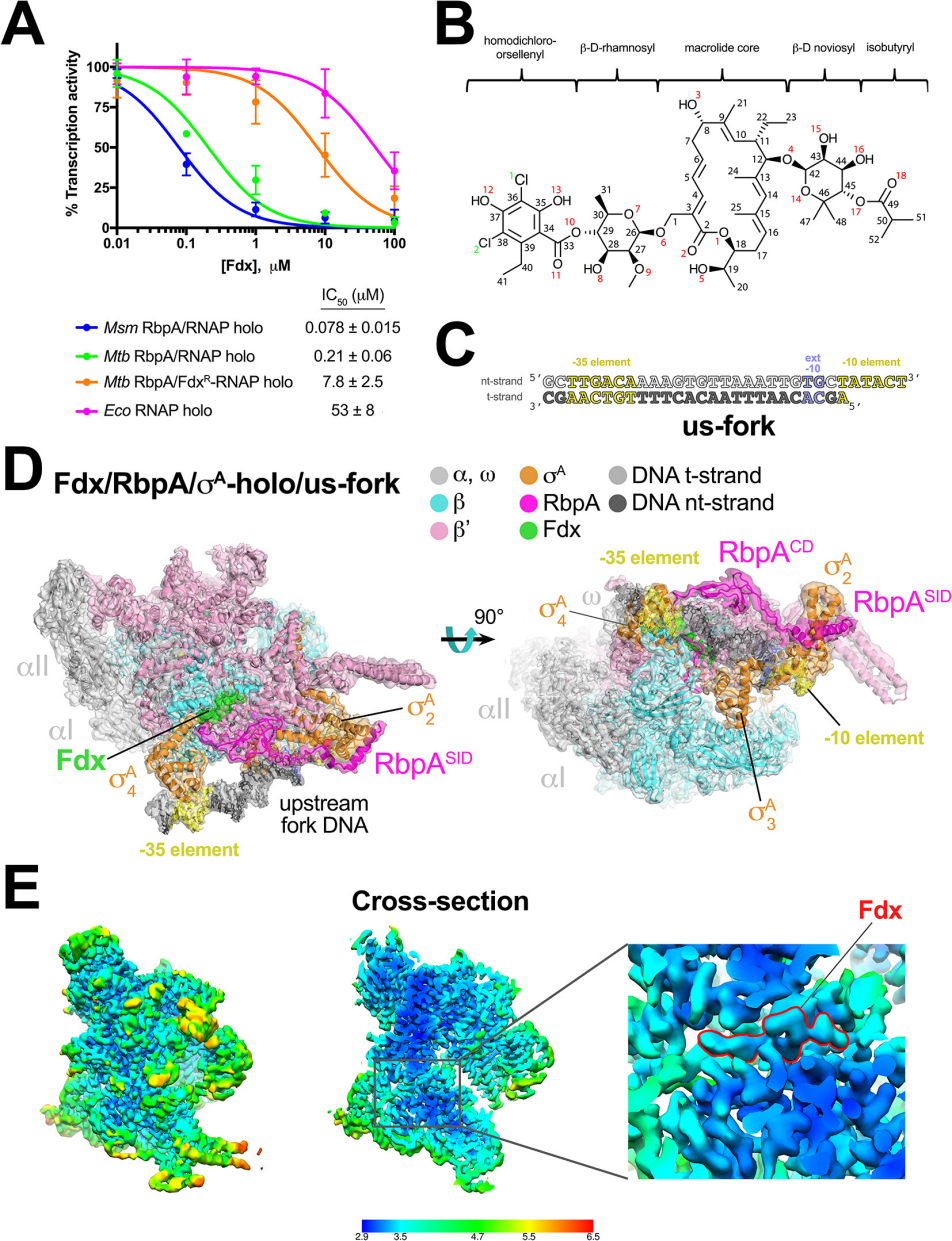
Structure of an *Mtb* RbpA/TIC with Fdx at 3.4 Å resolution. (**A**) Fdx inhibits mycobacterial RbpA/σ^A^-holo transcription greater than 250-fold more effectively than *Eco*σ^70^-holo in in vitro abortive initiation assays. The error bars denote the standard error from a minimum of three experiments (for some points, the error bars are smaller than the width of the point and are not shown). (**B**) Chemical structure of Fdx (Serra et al., 2017). (**C**) Synthetic us-fork promoter fragment used for cryo-EM experiments. The DNA sequence is derived from the full con promoter (Gaal et al., 2001). The nontemplate-strand DNA (top strand) is colored light gray; the template-strand DNA (bottom strand), dark grey. The −35 and −10 elements are shaded yellow. The extended −10 (Keilty and Rosenberg, 1987) is colored violet. (**D**) The 3.4 Å resolution cryo-EM density map of the Fdx/RbpA/σ^A^-holo/us-fork complex is rendered as a transparent surface colored as labeled. Superimposed is the final refined model; proteins are shown as a backbone ribbon, Fdx and the nucleic acids are shown in stick format. (**E**) Views of the cryo-EM map colored by local resolution based on blocres calculation (Cardone et al., 2013). The left view shows the entire map, while the middle view shows a cross-section of the map sliced at the level of the Fdx binding pocket. The boxed region is magnified on the right. Density for the Fdx molecule is outlined in red.

Fdx has potent inhibitory activity against multi-drug-resistant *Mtb* cells and the in vivo target is the RNAP (Kurabachew et al., 2008). To our knowledge, the in vitro activity of Fdx against mycobacterial RNAPs has not been reported. RbpA, essential in *Mtb*, is a component of transcription initiation complexes (TICs) that tightly binds the primary promoter specificity σ^A^ subunit of the RNAP holoenzyme (holo) (Bortoluzzi et al., 2013; Forti et al., 2011; Hubin et al., 2017a, 2015; Tabib-Salazar et al., 2013). We therefore compared Fdx inhibition of mycobacterial RNAPs containing core RNAP combined with σ^A^ (σ^A^-holo) and RbpA with inhibition of *Escherichia coli* (*Eco*) σ^70^-holo using a quantitative abortive initiation assay (Davis et al., 2015). Fdx inhibited *Mtb* and *M. smegmatis* (*Msm*) transcription at sub-μM concentrations, whereas inhibition of an *Mtb* TIC containing Fdx-resistant (Fdx^R^) RNAP (β^Q1054H^) (Kurabachew et al., 2008) required a nearly two orders of magnitude higher concentration of Fdx. *Eco* RNAP was inhibited even less effectively by another order of magnitude (Figure 1A, Figure 1—figure supplement 1A).

**Figure 1—figure supplement 1.**
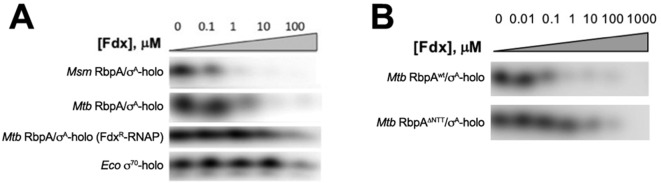
Abortive Transcription assays to determine IC_50_ for Fdx. (**A, B**) Abortive transcription activity of samples (labeled) after incubation with the indicated concentrations of Fdx.

**Figure 1—figure supplement 2.**
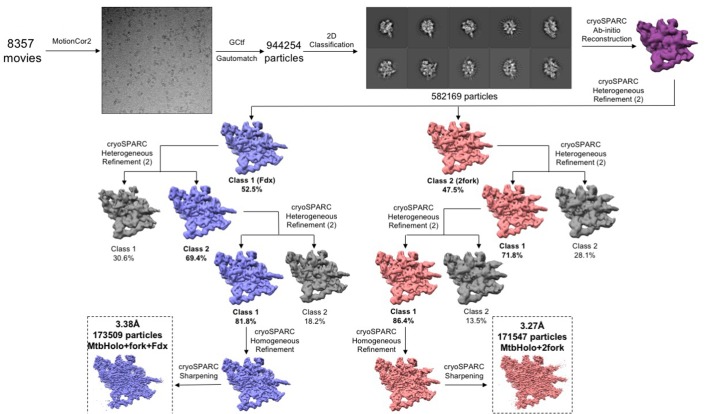
Data processing pipeline for the cryo-EM movies of the Fdx/RbpA/σ^A^-holo/us-fork complexes. Flowchart showing the image processing pipeline for the cryo-EM data of *Mtb* Fdx/RbpA/σ^A^-holo/us-fork complexes starting with 8357 dose-fractionated movies collected on a 300 keV Titan Krios (FEI) equipped with a K2 Summit direct electron detector (Gatan). Movies were frame aligned and summed using MotionCor2 (Zheng et al., 2017). CTF estimation for each micrograph was calculated with Gctf (Zhang, 2016). A representative micrograph is shown following processing by MotionCor2. Particles were autopicked from each micrograph with Gautomatch and then sorted by 2D classification using RELION (Scheres, 2012) to assess quality. The ten highest populated classes from the 2D classification are shown. After initial cleaning, the dataset contained 582,169 particles. A subset of particles was used to generate an *ab-initio* map in cryoSPARC (Punjani et al., 2017). Using the low-pass filtered (60 Å) *ab-initio* map as a template, 3D heterogeneous refinement was performed in cryoSPARC in a binomial-like fashion. Two major classes were observed: Fdx/RbpA/σ^A^-holo/us-fork (blue) and RbpA/σ^A^-holo//(us-fork)_2_ (red). Multiple rounds of 3D classification were performed; colored classes represent the selected classes while grey classes were thrown out. The final sets of particles for each class were refined using cryoSPARC homogenous refinement and then sharpened for model building.

**Figure 1—figure supplement 3.**
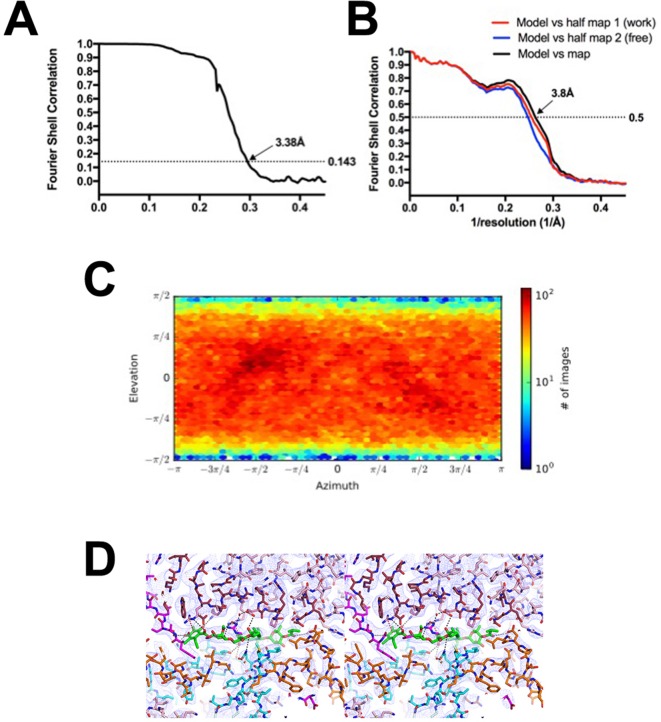
Fdx/RbpA/σ^A^-holo/us-fork class. (**A**) Gold-standard FSC (Rosenthal and Henderson, 2003), calculated by comparing the two independently determined half-maps from cryoSPARC. The dotted line represents the 0.143 FSC cutoff which indicates a nominal resolution of 3.38 Å. (**B**) FSC calculated between the refined structure and the half map used for refinement (work, red), the other half map (free, blue), and the full map (black). (**C**) Angular distribution calculated in cryoSPARC for particle projections. Heat map shows number of particles for each viewing angle (less = blue, more = red). (**D**) Stereo view of the Fdx binding pocket with cryo-EM density (blue mesh) superimposed.

### Cryo-EM structure of the Fdx/RbpA/σ^A^-holo complex

We used single-particle cryo-EM to examine the complex of *Mtb* RbpA/σ^A^-holo with and without Fdx (Figure 1B). Preliminary analyses revealed that the particles were prone to oligomerization, which was reduced upon addition of an upstream-fork (us-fork) junction promoter DNA fragment (Figure 1C). We sorted nearly 600,000 cryo-EM images of individual particles into two distinct classes, each arising from approximately half of the particles (Figure 1—figure supplement 2).

The first class comprised *Mtb* RbpA/σ^A^-holo with one us-fork promoter fragment and bound to Fdx. The cryo-EM density map was computed to a nominal resolution of 3.4 Å (Figure 1D, Figure 1—figure supplement 3, Supplementary file 1). The us-fork promoter fragment was bound outside the RNAP active site cleft, as expected, with the −35 and −10 promoter elements engaged with the σ^A^_4_ and σ^A^_2_ domains, respectively (Figure 1D). Local resolution calculations (Cardone et al., 2013) indicated that the central core of the structure, including the Fdx binding determinant and the bound Fdx, was determined to 2.9–3.4 Å resolution (Figure 1E).

### Cryo-EM structure of a *Mtb* RPo mimic

**Figure 2.**
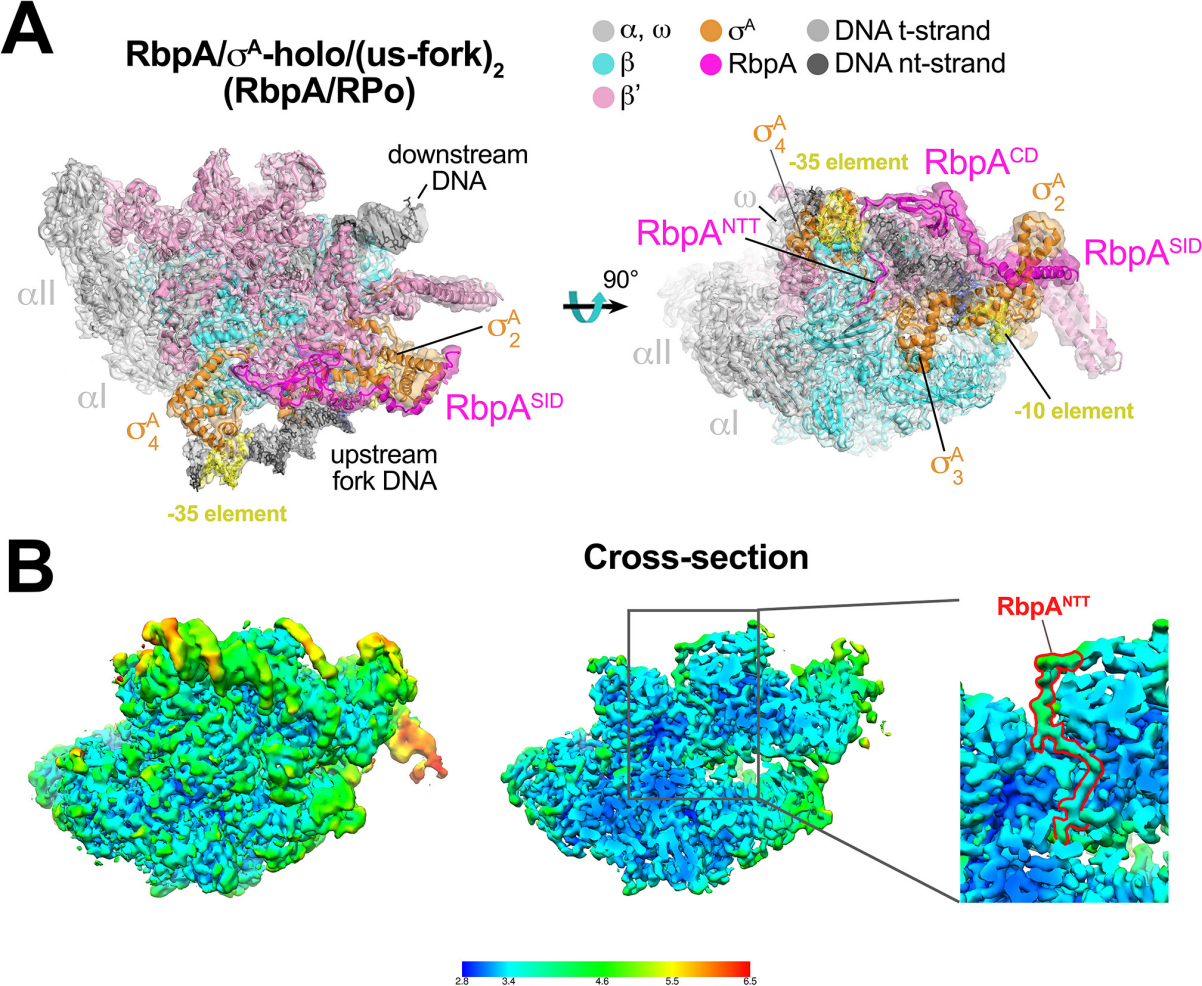
Structure of an *Mtb* RbpA/RPo mimic at 3.3 Å resolution. (**A**) The 3.3 Å resolution cryo-EM density map of the RbpA/σ^A^-holo/(us-fork)_2_ complex (RbpA/RPo mimic) is rendered as a transparent surface colored as labeled. Superimposed is the final refined model; proteins are shown as a backbone ribbon, nucleic acids are shown in stick format. (**B**) Views of the *Mtb* RbpA/RPo mimic cryo-EM map colored by local resolution based on blocres calculation (Cardone et al., 2013). The left view shows the entire map, while the middle view shows a cross-section of the map sliced at the level of the RbpA^NTT^. The boxed region is magnified on the right. Density for the RbpA^NTT^ is outlined in red.

The second class comprised *Mtb* RbpA/σ^A^-holo bound to two us-fork promoter fragments but without Fdx to a nominal resolution of 3.3 Å (Figure 2A, Figure 2—figure supplement 1, Supplementary file 1). One us-fork promoter fragment bound upstream from the RNAP active site cleft as in the previous class, but a second us-fork promoter fragment bound the RNAP downstream duplex DNA binding channel, with the 5-nucleotide 3'-overhang (Figure 1C) engaged with the RNAP active site (as the template strand) like previously characterized 3'-tailed templates (Gnatt et al., 2001; Kadesch and Chamberlin, 1982). Local resolution calculations (Cardone et al., 2013) indicated that the central core of the structure was determined to between 2.8–3.2 Å resolution (Figure 2B). The overall conformation of this protein complex and its engagement with the upstream and downstream DNA fragments was very similar to the crystal structure of a full *Msm* open promoter complex (RPo) (Hubin et al., 2017b) with one exception (see below). We will therefore call this complex an *Mtb* RbpA/RPo mimic.

**Figure 2—figure supplement 1.**
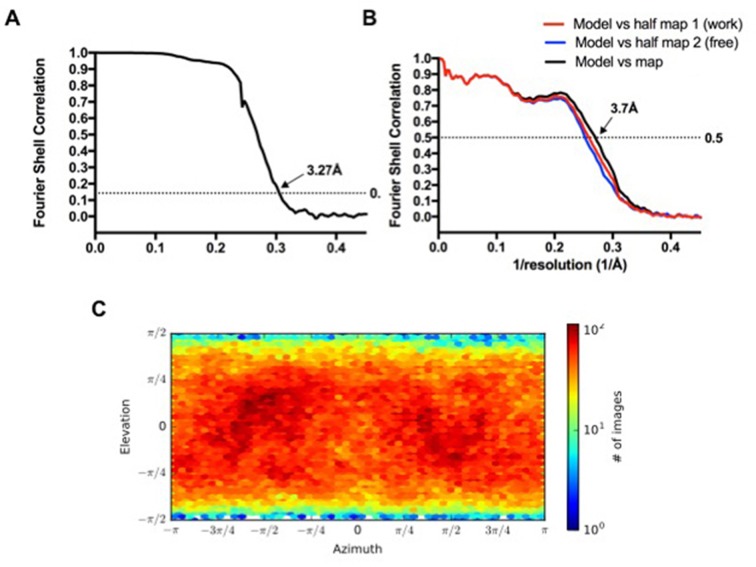
RbpA/σ^A^-holo/(us-fork)_2_ class. (**A**) Gold-standard FSC (Rosenthal and Henderson, 2003), calculated by comparing the two independently determined half-maps from cryoSPARC. The dotted line represents the 0.143 FSC cutoff which indicates a nominal resolution of 3.27 Å. (**B**) FSC calculated between the refined structure and the half map used for refinement (work, red), the other half map (free, blue), and the full map (black). (**C**) Angular distribution calculated in cryoSPARC for particle projections. Heat map shows number of particles for each viewing angle (less = blue, more = red).

### The RbpA N-terminal tail invades the RNAP active site cleft

**Figure 3.**
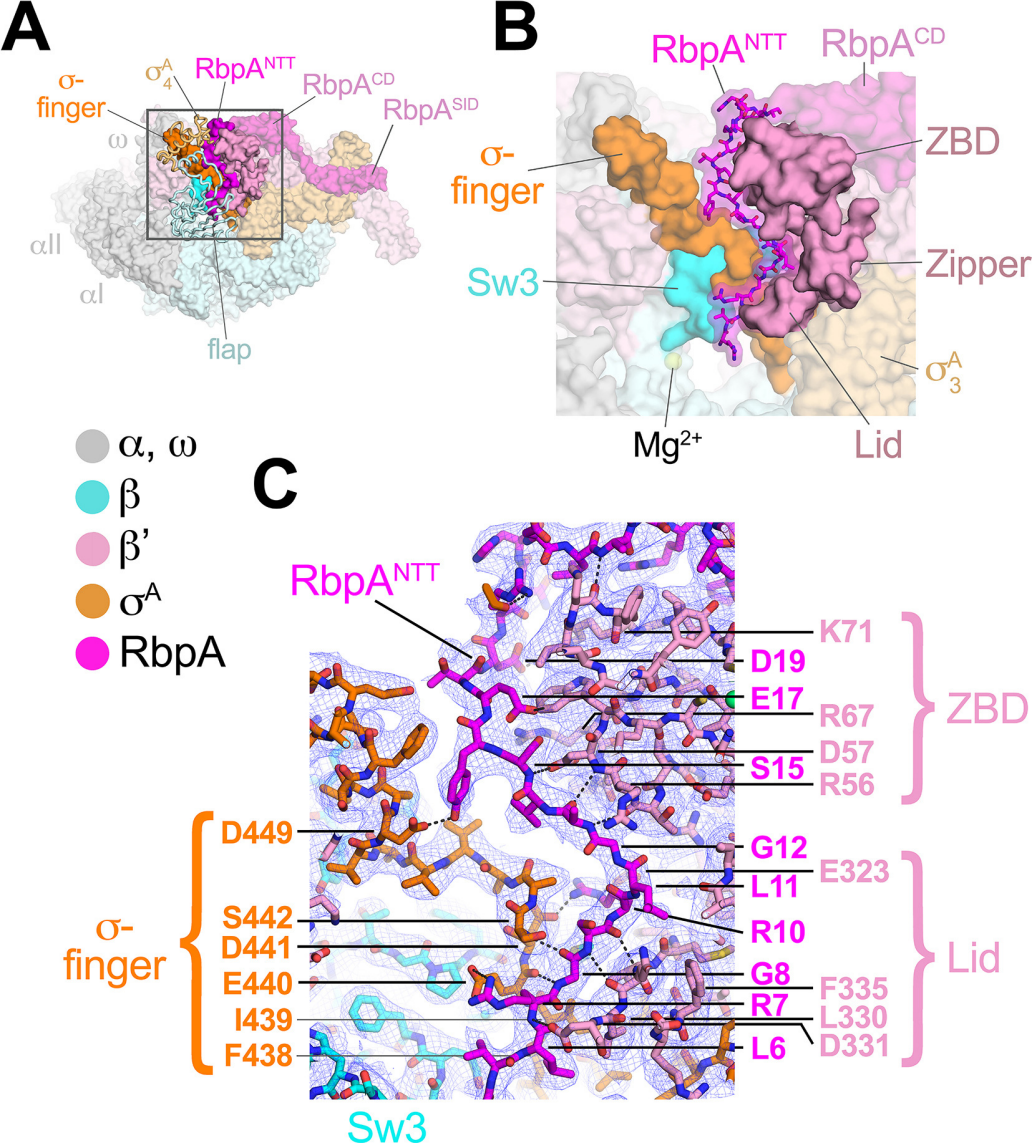
The RbpA^NTT^ interacts with conserved structural elements in the RNAP active site cleft. (**A**) An overview of the RbpA/RPo structure is shown as a color-coded molecular surface (color-coding denoted in the key) except the β flap and σ^A^_4_ domain are shown as backbone worms, revealing the RbpA^NTT^ (magenta) underneath. The DNA fragments are not shown. The boxed region is magnified in panel (**B**). (**B**) Magnified view of the boxed region from panel (**A**). The RbpA^NTT^ is shown in stick format with a transparent molecular surface. Conserved RNAP structural elements that interact with the RbpA^NTT^ are highlighted (βSw3, β'ZBD, β'Zipper, β'Lid, and σ-finger). (**C**) Further magnified view showing the cryo-EM density (blue mesh) with the superimposed model. Conserved residues of the RbpA^NTT^ are labeled, along with conserved residues of the β'ZBD, β'Lid, and σ-finger that interact with the RbpA^NTT^.

RbpA comprises four structural elements, the N-terminal tail (NTT), the core domain (CD), the basic linker, and the sigma interacting domain (SID) (Bortoluzzi et al., 2013; Hubin et al., 2017a; Tabib-Salazar et al., 2013). Our previous crystal structures of *Msm* TICs containing RbpA showed that the RbpA^SID^ interacts with the σ^A^_2_ domain, the RbpA^BL^ establishes contacts with the promoter DNA phosphate backbone just upstream of the −10 element, and the RbpA^CD^ interacts with the RNAP β' Zinc-Binding-Domain (ZBD) (Hubin et al., 2017a, 2017b). Density for the RbpA^NTT^ (RbpA residues 1–25) was never observed in the crystal structures and was presumed to be disordered. In striking contrast to the crystal structures, both cryo-EM structures reveal density for the RbpA^NTT^, which unexpectedly threads into the RNAP active site cleft between the ZBD and σ^A^_4_ domains and snakes through a narrow channel towards the RNAP active site Mg^2+^ (Figure 3). On its path, conserved residues of the RbpA^NTT^ interact with conserved residues of the σ-finger (σ_3.2_-linker) on one wall of the channel, and with conserved residues of the ZBD and β'lid on the other wall (Figure 3C).

The most N-terminal RbpA residues visible in the cryo-EM structures (A2 in the Fdx complex, R4 in the RPo) sit near the tip of the σ-finger where it makes its closest approach to the RNAP active site, too far (25 Å) to play a direct role in RNAP catalytic activity or substrate binding. The σ-finger plays an indirect role in transcription initiation, stimulating de novo phosphodiester bond formation by helping to position the t-strand DNA (Kulbachinskiy and Mustaev, 2006; Zhang et al., 2012). The σ-finger is also a major determinant of abortive initiation, playing a direct role in initiation and promoter escape by physically blocking the path of the elongating RNA transcript before σ release (Cashel et al., 2003; Murakami et al., 2002). The intimate association of the RbpA^NTT^ with the σ-finger (Figure 3C) suggests that the RbpA^NTT^ also plays a role in these processes of *Mtb* RNAP initiation. This is consistent with our findings that the RbpA^NTT^ does not strongly affect RPo formation but plays a significant role in in vivo gene expression in *Msm* (Hubin et al., 2017a). This location of the RbpA^NTT^ explains the high Fdx sensitivity of *Mtb* RNAP (see below).

### Fdx interacts with RNAP, σ^A^, and RbpA

**Figure 4.**
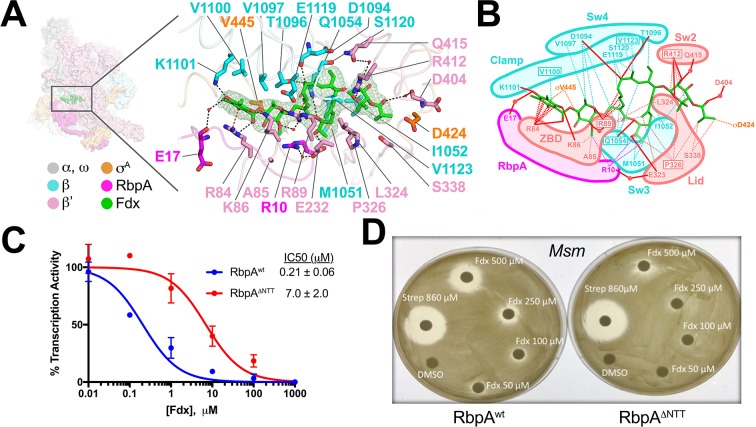
Structural basis for Fdx inhibition of *Mtb* transcription and the role of the RbpA^NTT^. (**A**) (left) Overview of the Fdx/RbpA/σ^A^-holo/us-fork structure, shown as a molecular surface (the DNA is not shown). The boxed region is magnified on the right. (right) Magnified view of the Fdx binding pocket at the same orientation as the boxed region on the left. Proteins are shown as α-carbon backbone worms. Residues that interact with Fdx are shown in stick format. Fdx is shown in stick format with green carbon atoms. Water molecules are shown as small pink spheres. Hydrogen-bonds are indicated by dashed gray lines. Cation-π interactions (between β'R84 and the aromatic ring of the Fdx homodichloroorsellinic acid moiety and β'R89 and the conjugated double-bond system centered between C4 and C5 of the macrolide core) are represented by red dashed lines. (**B**) Schematic summary of the Fdx contacts with σ^A^-holo and RbpA. Fdx is shown in stick format with green carbon atoms. Thin dashed lines represent van der Waals contacts (≤4.5 Å), thick red lines represent hydrogen bonds (<4 Å). The thin dashed red lines denote cation-π interactions. (**C**) The RbpA^NTT^ is required for optimal inhibition of *Mtb* transcription by Fdx in in vitro abortive initiation assays. The error bars denote the standard error from a minimum of three experiments (for some points, the error bars are smaller than the width of the point and are not shown). (**D**) Zone of inhibition assays with *Msm* cells show that loss of the RbpA-NTT (RbpA^ΔNTT^) leads to loss of Fdx sensitivity in vivo.

The reconstruction from the Fdx-bound class (Figure 1D) reveals unambiguous density for Fdx (Figure 4A) and defines Fdx-interacting residues from four protein components of the complex, β, β', σ^A^, and RbpA, including six water molecules, four of which mediate Fdx/RNAP interactions (Figure 4A,B). Fdx binding to the TIC buries a large accessible surface area of 4,800 Å^2^ (β, 2,100 Å^2^; β', 2,000 Å^2^; σ^A^, 300 Å^2^; RbpA, 330 Å^2^). Fdx forms direct hydrogen bonds with nine residues (βQ1054, βD1094, βT1096, βK1101, β'R84, β'K86, β'R89, β'E323, and β'R412) and water-mediated interactions with four (β'R89, β'D404, β'Q415, and RbpA-E17). Notably, the Fdx/RNAP interaction is stabilized by two cation-π interactions between β'R84 and the aromatic ring of the Fdx homodichloroorsellinic acid moiety (Figure 1B) and β'R89 and the conjugated double-bond system centered between C4 and C5 of the macrolide core (Figures 1B and 4A,B). Fdx interacts with residues from eight distinct structural elements (Lane and Darst, 2010) of the initiation complex (βSw3, βSw4, β residues belonging to the clamp, β'ZBD, β'lid, β'Sw2, the σ-finger, and the Rbpa^NTT^ (Figure 4A,B).

Amino-acid substitutions conferring Fdx^R^ have been identified in RNAP β or β' subunits from *Bacillus subtilis* (Gualtieri et al., 2006), *Cdf* (Kuehne et al., 2017), *Enterococcus faecalis* (Gualtieri et al., 2009), and *Mtb* (Kurabachew et al., 2008), corresponding to *Mtb* RNAP β residues Q1054 (Sw3), V1100 and V1123 (clamp), and β' residues R89 (ZBD), P326 (lid), and R412 (Sw2). The structure shows that each of these residues makes direct interactions with Fdx (Figure 4A,B). All five chemical moieties of Fdx (Figure 1B) interact with at least one RNAP residue that confers Fdx^R^ when mutated (Figure 4B), suggesting that each moiety may be important for Fdx action.

### The RbpA^NTT^ is critical for Fdx potency against mycobacterial RNAP in vitro and in vivo

In addition to the β and β' subunits, Fdx interacts with residues of the σ-finger (D424 and V445; Figure 4A,B). Finally and unexpectedly, Fdx contacts residues from the RbpA^NTT^ (Figure 4A,B). To test the functional importance of the RpbA^NTT^ for Fdx inhibition in vitro, we compared Fdx inhibition of *Mtb*σ^A^-holo with either RbpA or RbpA with the NTT truncated (RbpA^ΔNTT^) in the abortive initiation assay (Figure 1—figure supplement 1B). Truncation of the RbpA-NTT caused a 35-fold increase in resistance to Fdx (Figure 4C).

RbpA is essential in *Mtb* and *Msm*, but strains carrying RbpA^ΔNTT^ are viable (Hubin et al., 2017a), allowing us to test the role of the RbpA^NTT^ in Fdx growth inhibition of *Msm* cells. We performed zone of inhibition assays on two *Msm* strains that are isogenic except one harbors wild-type RbpA (RbpA^wt^) and the other RbpA^ΔNTT^ (Hubin et al., 2017a). The *Msm* RbpA^∆NTT^ strain grew considerably slower on plates, taking approximately twice the time as the wild-type *Msm* to reach confluency. Despite the growth defect, the RbpA^ΔNTT^ strain was significantly less sensitive to Fdx (Figure 4D). Discs soaked with up to 250 μM Fdx did not produce inhibition zones with RbpA^ΔNTT^ but inhibition zones were apparent with RbpA^wt^. At 500 μM Fdx, the inhibition zone for RbpA^ΔNTT^ was significantly smaller than for RbpA^wt^. By contrast, 860 μM streptomycin, a protein synthesis inhibitor, produced equal inhibition zones for the RbpA^wt^ and RbpA^ΔNTT^ strains. We conclude that the essential role of RbpA in *Mtb* transcription is key to the relatively high sensitivity of *Mtb* cells to Fdx.

### Fdx traps an open-clamp conformation

The RNAP switch regions are thought to act as hinges connecting the mobile clamp domain to the rest of the RNAP (Gnatt et al., 2001; Lane and Darst, 2010). Bacterial RNAP inhibitors myxopyronin, corallopyronin, and ripostatin bind Sw1 and Sw2 and stabilize a closed-clamp conformation of the RNAP (Belogurov et al., 2009; Mukhopadhyay et al., 2008). The Fdx binding determinant does not overlap the sites for these other inhibitors, but the Fdx interactions with the Sw2, Sw3, and Sw4 regions (Figure 4A,B) suggest that Fdx may influence the clamp conformation as well.

**Figure 5.**
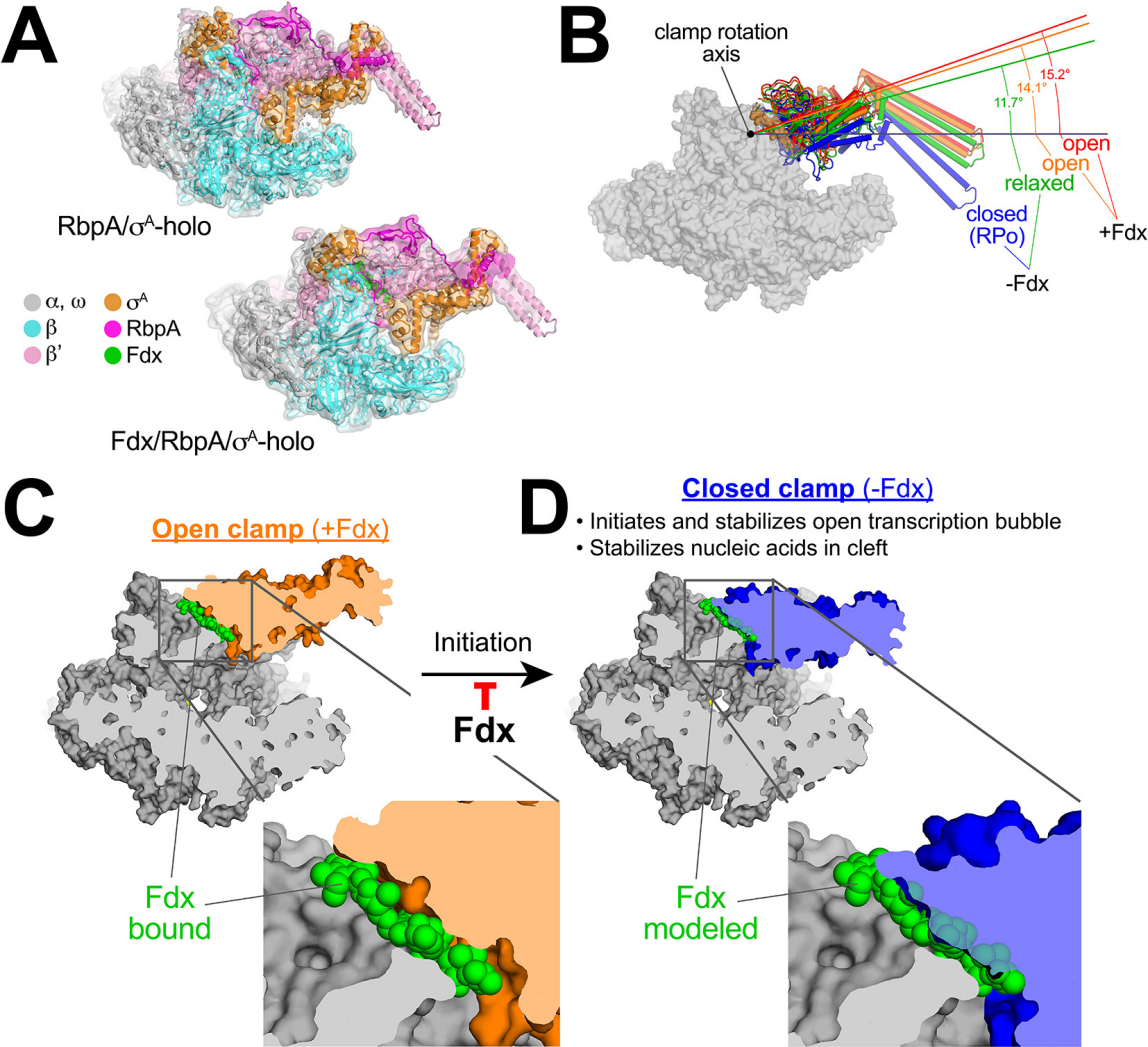
Mechanism of Fdx inhibition of bacterial RNAP. (**A**) Cryo-EM density maps and superimposed refined models for *Mtb* RbpA/σ^A^-holo (5.2 Å resolution) and *Mtb* Fdx/RbpA/σ^A^-holo (6.5 Å resolution). (**B**) RNAP clamp conformational changes for four cryo-EM structures determined in this work. The RbpA/RPo (Figure 2A) structure was used as a reference to superimpose the other structures via α-carbon atoms of the structural core module (Supplementary file 2), revealing a common core RNAP structure (shown as a gray molecular surface) but with large shifts in the clamp modules. The clamp modules are shown as backbone cartoons with cylindrical helices and color-coded (blue, closed clamp of RPo; green, relaxed clamp of RbpA/σ^A^holo; orange, open clamp of Fdx/RbpA/σ^A^-holo/us-fork; red, open clamp of Fdx/RbpA/σ^A^-holo). The clamp conformational changes can be characterized as rigid body rotations about a rotation axis perpendicular to the page (denoted). The angles of clamp opening for the different structures are shown (relative to the blue closed RPo clamp, 0° opening). (**C**) The core RNAP from the 3.4 Å resolution Fdx/RbpA/σ^A^-holo/us-fork structure is shown as a gray molecular surface but with the open clamp colored orange. The structure is sliced at the level of the Fdx binding pocket (the bound Fdx is shown in green). The boxed region is magnified below, showing the tight fit of the Fdx molecule in a narrow gap between the clamp and the rest of the RNAP. (**D**) The core RNAP from the 3.3 Å resolution RbpA/RPo structure is shown as a gray molecular surface but with the closed clamp colored blue. The structure is sliced at the level of the (empty) Fdx binding pocket. Fdx, modeled from the structure shown in (**C**), is shown in green. The boxed region is magnified below. Fdx cannot bind to RNAP with a closed clamp because clamp closure pinches off the Fdx binding site. Clamp closure is required for initiation and stabilization of the transcription bubble (Feklistov et al., 2017) and also for stable binding of nucleic acids in the RNAP cleft.

To understand the role of Fdx in clamp movement without the complication of DNA binding in the RNAP active site cleft, we determined cryo-EM structures of *Mtb* RbpA/σ^A^-holo without DNA, with Fdx and without Fdx. Although the particles in the original cryo-EM datasets of *Mtb* RbpA/σ^A^-holo were prone to oligomerization, we used 2D classification to isolate single particles and determined reconstructions of *Mtb* RbpA/σ^A^-holo without DNA and with Fdx (overall 6.5 Å resolution from 21,000 particles, Figure 5A, Figure 5—figure supplement 1A–E) and without Fdx (overall 5.2 Å resolution from 88,000 particles; Figure 5A, Figure 5—figure supplement 1F–I). The cryo-EM density maps were of sufficient detail to visualize the bound antibiotic in the Fdx complex (Figure 5—figure supplement 1E) and to determine the domain organization (including the clamp conformation) by rigid-body refinement (Figure 5A). Thus, we were able to compare the RNAP conformational states from four solution complexes of the same RNAP in the absence of crystal packing forces (Figure 5B).

**Figure 5—figure supplement 1.**
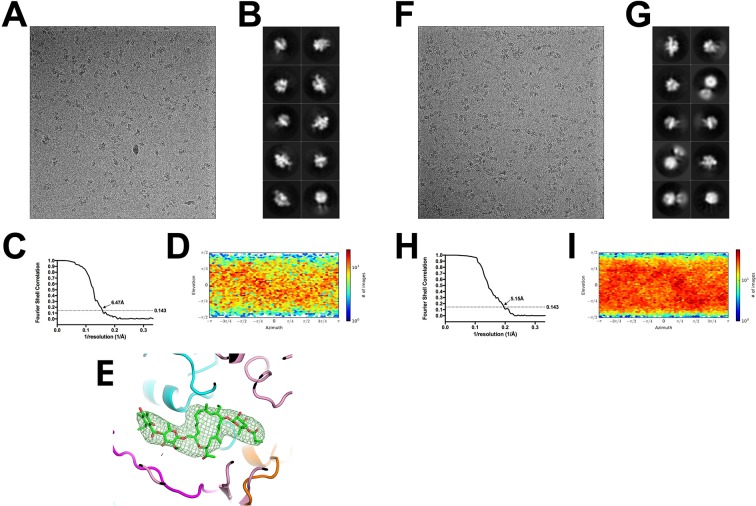
Cryo-EM of the *Mtb* RbpA/σ^A^-holo and Fdx/RbpA/σ^A^-holo complexes. (**A**) Representative micrograph of the Fdx/RbpA/σ^A^-holo complex in vitreous ice. (**B**) The ten highest populated classes from cryoSPARC 2D classification. (**C**) Gold-standard FSC of the *Mtb* Holo/Fdx complex. The gold-standard FSC was calculated by comparing the two independently determined half-maps from cryoSPARC. The dotted line represents the 0.143 FSC cutoff which indicates a nominal resolution of 6.47 Å. (**D**) Angular distribution calculated in cryoSPARC for *Mtb* Fdx/RbpA/σ^A^-holo particle projections. Heat map shows number of particles for each viewing angle (less = blue, more = red). (**E**) Difference map showing Fdx is bound in 6.5 Å resolution structure. (**F**) Representative micrograph of the *Mtb* RbpA//σ^A^-holo complex in vitreous ice. (**G**) The ten highest populated classes from cryoSPARC 2D classification. (**H**) Gold-standard FSC of the *Mtb* Holo complex. The gold-standard FSC was calculated by comparing the two independently determined half-maps from cryoSPARC. The dotted line represents the 0.143 FSC cutoff which indicates a nominal resolution of 5.15 Å. (**I**) Angular distribution calculated in cryoSPARC for *Mtb* Holo particle projections. Heat map shows number of particles for each viewing angle (less = blue, more = red).

The four structures were superimposed by the structural core module (Supplementary file 2), comprising the ω subunit and highly conserved β and β′ regions in or near the active center that have not been observed to undergo significant conformational changes in dozens of RNAP structures. Using the RPo structure (Figure 2A) as a reference, the structures superimposed with rmsds < 0.4 Å over at least 898 aligned α-carbon (Cα) atoms of the structural core module, but rmsds > 9 Å for 461 Cα-atoms of the clamp modules (Supplementary file 2), indicating large shifts of the clamp module with respect to the rest of the RNAP in the different complexes.

Alignment of the structures revealed that the clamp conformational changes could be characterized as rigid body rotations about a common rotation axis (Figure 5B). Assigning a clamp rotation angle of 0° (closed clamp) to the RPo structure (blue, Figure 5B), the RbpA/σ^A^-holo clamp is rotated open by about 12° (green, Figure 5B). Because this complex is not interacting with any other ligands that might be expected to alter the clamp conformation (such as Fdx or DNA), we will refer to this as the 'relaxed' clamp conformation. The two Fdx-bound complexes, with or without us-fork DNA, show further opening of the clamp (14° and 15°, respectively; orange and red in Figure 5B).

### Fdx acts like a doorstop to stabilize the open-clamp conformation

In the high-resolution Fdx/TIC structure (Figure 1D), Fdx binds in a narrow gap between the open clamp module and the rest of the RNAP (Figure 5C). Examination of the high-resolution RPo (closed clamp) structure reveals that clamp closure pinches off the Fdx binding pocket (Figure 5D) - Fdx can only bind to the open-clamp conformation of RNAP. We thus conclude that Fdx acts like a doorstop, binding and stabilizing the open-clamp conformation.

## Discussion

### Fdx inhibits RNAP by trapping an open-clamp conformation

Clamp dynamics play multiple important roles in the transcription cycle. Motions of the clamp module and the role of the switch regions as hinges were first noted by comparing crystal structures of free RNAPs (Cramer et al., 2001; Zhang et al., 1999) with the crystal structure of an elongation complex containing template DNA and RNA transcript (Gnatt et al., 2001). Binding of the downstream duplex DNA and RNA/DNA hybrid in the RNAP active-site cleft was proposed to close the clamp around the nucleic acids, explaining the high processivity of the transcription elongation complex. Numerous subsequent crystal structures have supported the idea that stable, transcription-competent complexes of RNAP with nucleic acids, either RPo (Bae et al., 2015; Hubin et al., 2017b; Zuo and Steitz, 2015) or elongation complexes (Gnatt et al., 2001; Kettenberger et al., 2004; Vassylyev et al., 2007), correlate with the closed-clamp conformation. Effects of crystal packing forces on clamp conformation, however, cannot always be ruled out. Observations of clamp positions by solution FRET (Chakraborty et al., 2012), and more recently in cryo-EM structures (Bernecky et al., 2016; Hoffmann et al., 2015; Kang et al., 2017; Neyer et al., 2016) (in the absence of crystal packing forces) have confirmed the relationship between clamp closure and stable nucleic-acid complexes. Clamp motions have also been shown to play a critical role in the process of promoter melting to form the transcription bubble during RPo formation (Feklistov et al., 2017). Thus, the trapping of an open-clamp RNAP conformation by Fdx in unrestrained cryo-EM conditions (Figure 5C) suggests that Fdx inhibits transcription initiation by preventing clamp motions required for RPo formation, or by not allowing RNAP to form stable transcription-competent complexes with nucleic acids, or both (Figure 5C,D). These results are broadly consistent with mechanistic analyses of (Tupin et al., 2010) and (Morichaud et al., 2016) showing that Fdx blocks promoter melting at an early step but providing RNAP a pre-melted template overcomes the block. These authors proposed that Fdx likely prevented the clamp from closing, again consistent with our structural findings.

### Summary

Our results establish the molecular details of Fdx interactions with the bacterial RNAP (Figure 4A,B) and a mechanism of action for Fdx (Figure 5C,D). Crucially, the essential actinobacterial GTF RbpA is responsible for the high sensitivity of mycobacterial RNAP to Fdx both in vitro (Figure 4C) and in vivo (Figure 4D). This new knowledge provides a structural platform for the development of antimicrobials that target the Fdx binding determinant and underscores the need to define structure-activity relationships of drug leads using near-native states, in this case using cryo-EM with the RbpA/σ^A^-holo complex to guide development of effective *Mtb* treatments.

## Materials and methods

**Key resources table:** 

Reagent type (species) or resource	Designation	Source or reference	Identifiers	Additional information
Strain, strain background (*Escherichia coli*)	*Eco* BL21(DE3)	EMD-Millipore (Novagen; Darmstadt, Germany)		
Strain, strain background (*Mycobacterium* *smegmatis* mc2155)	MGM6029: *Msm* mc2155 *rpoC:rpoC-* *ppx-10his hyg*	PMID: 28067618		
Strain, strain background (*Mycobacterium* *smegmatis* mc2155)	MGM6234: *Msm* mc2155*ΔrbpA attB::* *rbpA(28-114) kan*	PMID: 28067618		
Recombinant DNA reagent	pAC22	PMID: 24713321		pET28a derivative. Encodes M. bovis RNAP. β contains a S450Y substitution (RifR) and a short N-terminal insertion at codon 2 (LEGCIL); β′ has C-terminal His8 tag; β and β′ are fused with a short linker (LARHGGSGA)
Recombinant DNA reagent	pACYCDuet- 1_Ec_rpoZ	PMID: 23389035		
Recombinant DNA reagent	pET21a-*Eco*σ70	PMID: 24218560		
Recombinant DNA reagent	pet21C-*Msm*RbpA	PMID: 28067618		
Recombinant DNA reagent	pet21C-*Mtb*RbpA	PMID: 28067618		
Recombinant DNA reagent	pET28a	EMD-Millipore (Novagen)		
Recombinant DNA reagent	pET-SUMO *Msm*σA	PMID: 28067618		
Recombinant DNA reagent	pET-SUMO *Mtb*σA	PMID: 28067618		
Recombinant DNA reagent	pET-SUMO *Msm*RbpAΔNTT	PMID: 28067618		
Recombinant DNA reagent	pET-SUMO *Mtb*RbpAΔNTT	PMID: 28067618		
Recombinant DNA reagent	pGEMABC	PMID: 23389035	Addgene 45398	
Recombinant DNA reagent	pMP55	this paper		pAC22 derivative encoding Mtb RNAP with β S450Y. Derived from pAC22 by P69R substitution, removal of the N-terminal β insertion, and substitutions of increased predicted-strength RBSs for the rpoA, rpoZ, and rpoB::C RBSs.
Recombinant DNA reagent	pMP57	this paper		pMP55 with β Q1054H. Fdx resistant.
Recombinant DNA reagent	pMP61	this paper		pMP55 with wild-type S450 in place of β Y450.
Recombinant DNA reagent	pMP62	this paper		pMP61 with β S450L.
Recombinant DNA reagent	pRARE2	EMD-Millipore (Novagen)		
Recombinant DNA reagent	pUC57-AP3	PMID: 25510492		
Chemical compound, drug	Fidaxomicin	VWR International, Inc. (Radnor, PA)	2832–1	
Chemical compound, drug	3-([3-cholamidopropyl]dimethylammonio)−2 -hydroxy-1-propanesulfonate (CHAPSO)	Sigma-Aldrich (St. Louis, MO)	C4695	
Software, algorithm	Blocres	PMID: 23954653		
Software, algorithm	Chimera	PMID: 15264254		
Software, algorithm	Coot	PMID: 15572765		
Software, algorithm	CryoSPARC	PMID: 28165473		
Software, algorithm	EMAN2	PMID: 16859925		
Software, algorithm	Gautomatch	http://www.mrc-lmb.cam.ac.uk/kzhang/Gautomatch		
Software, algorithm	Gctf	PMID: 26592709		
Software, algorithm	Leginon	PMID: 20817100		
Software, algorithm	Molprobity	PMID: 20057044		
Software, algorithm	MotionCor2	PMID: 28250466		
Software, algorithm	Phenix	PMID: 20124702		
Software, algorithm	PyMOL	Schrödinger, LLC (New York, NY)	http://www.pymol.org	
Software, algorithm	RELION	PMID: 23000701		
Software, algorithm	Serial EM	PMID: 16182563		
Software, algorithm	Unblur	PMID: 26023829		
Other	C-flat CF-1.2/1.3 400 mesh gold grids	Electron Microscopy Sciences (Hatfield, PA)	CF413-100-Au	

### Protein expression and purification

Mtb *RNAP overexpression plasmid.* The overexpression plasmid (OEP) for *Mtb* RNAP was engineered from an existing OEP for *M. bovis* RNAP (Czyz et al., 2014), pAC22. Four modifications were made. First, a sequence in pAC22 that encodes an N-terminal 6-aa insertion at codon 2 of *rpoB* was removed. Second, a sequence upstream of *rpoZ* that included an ATG that potentially allowed an N-terminal extension on ω was removed. Third, to increase protein expression, the ribosome-binding sites (RBSs) for *rpoA*, *rpoZ*, and *rpoB::C* were re-engineered to encode stronger predicted RBSs using predicted translation initiation rates calculated using the Salis RBS strength calculator (https://salislab.net/software/) (Espah Borujeni et al., 2014). Finally, the single amino-acid difference between *Mtb* RNAP and *Mbo* RNAP at position 69 of β was changed from Pro (*Mbo*) to Arg (*Mtb*) (P69R). The resulting plasmid, pMP55, encodes β S450Y (Rif^R^) *Mtb* RNAP. A wild-type derivative (Rif^S^) was engineered by site-direct mutagenesis to give plasmid pMP61 that expresses the wild-type *Mtb* RNAP. A derivative of pMP55 encoding the β Q1054H substitution that confers resistance to Fidaxomicin (Fdx) (Kurabachew et al., 2008) was constructed by site-directed mutagenesis.

Samples for Cryo-EM grid preparation used *Mtb* His-tagged-σ^A^ and RbpA co-expressed and purified as previously described (Hubin et al., 2015; 2017a). To compare Fdx sensitivity of full-length RbpA and RbpA^ΔNTT^, these proteins, and *Mtb* His-tagged-σ^A^ were expressed separately and purified as described previously (Hubin et al., 2015; 2017a). Briefly, Rosetta-2 cells (EMD-Millipore/Novagen) were co-transformed with pET plasmids expressing *Mtb* σ^A^(His-tagged) and RbpA and induced with 0.5 mM IPTG at 30°C for 4 hr. Clarified lysates was subjected to Ni^2+^ affinity, removal of the His-tag, a second Ni^2+^ affinity (collecting the flow through this time) and size exclusion chromatography.

*Mtb* RNAP was expressed and purified as previously described for *Mbo* and *Msm* RNAPs (Davis et al., 2015; Hubin et al., 2017a). *Eco* core RNAP, *Eco* σ^70^, *Msm* σ^A^, *Msm* RbpA, and *Msm* core RNAP were expressed and purified as described (Davis et al., 2015; Hubin et al., 2015; 2017a).

### In vitro transcription assays

In vitro abortive initiation transcription assays were performed using the WT AP3 (−87 to +71) promoter at 37°C as described (Davis et al., 2015): Assays were performed in KCl assay buffer (10 mM Tris-HCl, pH 8.0, 50 mM KCl, 10 mM MgCl_2_, 0.1 mM EDTA, 0.1 mM DTT, 50 μg-/mL BSA). The IC_50_'s of Fdx on the different holos were calculated as follows: *Mtb* and *Msm* RNAP holo were incubated with the cognate σ^A^ and RbpA-FL or RbpA^∆NTT^, and *Eco* RNAP (50 nM) was incubated with σ^70^, to form holos. Holos were incubated with Fdx for 10 min at 37°C prior to addition of template DNA. DNA template was added (10 nM final) and the samples were incubated for 15 min at 37°C for open complex formation. Transcription was initiated with nucleotide mix, and stopped with a 2X Stop buffer (45 mM Tris-HCl, 45 mM Boric acid, 8 M Urea, 30 mM EDTA, 0.05% bromophenol blue, 0.05% xylene cyanol) after 10 min at 37°C. Transcription products were denatured by heating at 95°C for two minutes and visualized by polyacrylamide gel electrophoresis using phosphorimagery and quantified using ImageJ (Schneider et al., 2012).

### Agar disk diffusion assay

*Msm* strains MGM6232 (ΔrbpA attB::rbpA kan) and MGM6234 (ΔrbpA attB::rbpA(28-114) kan) (Hubin et al., 2017a) were grown overnight in LBsmeg (LB with 0.5% glycerol, 0.5% dextrose and 0.05% Tween_80_) and 2 mL were centrifuged and resuspended in 200 µL of residual media and then plated. Filter discs were placed on the plates and stock solutions of Fdx were prepared in 10% DMSO at different concentrations (50 μM, 100 μM, 250 μM and 500 μM). 10 μl of antibiotic from each stock solution was pipetted onto the disks. Streptomycin (0.5 mg/ml, 860 μM) and 10% DMSO were used as positive and negative controls, respectively. Plates were incubated at 37°C for 74 hr and the zone of inhibition around each disk was photographed and measured.

### Preparation of Fdx/RbpA/σ^A^-holo Complexes for Cryo-EM

*Mtb* RbpA/σ^A^-holo (0.5 ml of 5 mg/ml) was injected into a Superose 6 Increase column (GE Healthcare Life Sciences, Pittsburgh, PA) equilibrated with 20 mM Tris-HCl pH 8.0, 150 mM K-Glutamate, 5 mM MgCl_2_, 2.5 mM DTT. The peak fractions of the eluted protein were concentrated by centrifugal filtration (EMD-Millipore, Darmstadt, Germany) to 6 mg/mL protein concentration. Fdx (when used) was added at 100 μM and us-fork DNA (when used) was added to 20 μM. The samples were incubated on ice for 15 min and then 3-([3-cholamidopropyl]dimethylammonio)−2-hydroxy-1-propanesulfonate (CHAPSO) was added to the sample for a final concentration of 8 mM prior to grid preparation.

### Cryo-EM grid preparation

C-flat CF-1.2/1.3-4Au 400 mesh gold grids (Protochips, Morrisville, NC) were glow-discharged for 20 s prior to the application of 3.5 μl of the sample (4.0–6.0 mg/ml protein concentration). After blotting for 3–4.5 s, the grids were plunge-frozen in liquid ethane using an FEI Vitrobot Mark IV (FEI, Hillsboro, OR) with 100% chamber humidity at 22°C.

### Cryo-EM data acquisition and processing

Structural biology software was accessed through the SBGrid consortium (Morin et al., 2013).

*Fdx/RbpA/σ^A^-holo/us-fork.* The grids were imaged using a 300 keV Titan Krios (FEI) equipped with a K2 Summit direct electron detector (Gatan, Warrendale, PA). Images were recorded with Leginon (Nicholson et al., 2010) in counting mode with a pixel size of 1.1 Å and a defocus range of 0.8 μm to 1.8 μm. Data were collected with a dose of 8 electrons/px/s. Images were recorded over a 10 s exposure with 0.2 s frames (50 total frames) to give a total dose of 66 electrons/Å^2^. Dose-fractionated subframes were aligned and summed using MotionCor2 (Zheng et al., 2017) and subsequent dose-weighting was applied to each image. The contrast transfer function was estimated for each summed image using Gctf (Zhang, 2016). From the summed images, Gautomatch (developed by K. Zhang, MRC Laboratory of Molecular Biology, Cambridge, UK, http://www.mrc-lmb.cam.ac.uk/kzhang/Gautomatch) was used to pick particles with an auto-generated template. Autopicked particles were manually inspected, then subjected to 2D classification in cryoSPARC (Punjani et al., 2017) specifying 50 classes. Poorly populated and dimer classes were removed, resulting in a dataset of 582,169 particles. A subset of the dataset was used to generate an initial model of the complex in cryoSPARC (*ab-initio* reconstruction). Using the *ab-initio* model (low-pass filtered to 30 Å-resolution), particles were 3D classified into two classes using cryoSPARC heterogenous refinement. CryoSPARC homogenous refinement was performed for each class using the class map and corresponding particles, yielding two structures with different clamp conformations: open (Fdx/RbpA/σ^A^-holo/us-fork; Figure 1D) and closed [RbpA/σ^A^-holo/(us-fork)_2_; Figure 2A]. Two rounds of heterogenous/homogeneous refinements were performed for each class to achieve the highest resolution. The open class (Fdx/RbpA/σ^A^-holo/us-fork) contained 173,509 particles with an overall resolution of 3.38 Å (Figure 1—figure supplement 3A) while the closed class [*Mtb* RNAP/σ^A^/RbpA/(us-fork)_2_] contained 171,547 paricles with a overall resolution of 3.27 Å (Figure 2—figure supplement 1A). Particle orientations of each class were plotted in cryoSPARC (Figure 1—figure supplement 1C, Figure 2—figure supplement 1C). FSC calculations (Figure 1—figure supplement 1A, Figure 2—figure supplement 1A) were performed in cryoSPARC and the half-map FSC (Figure 1—figure supplement 1B, Figure 2—figure supplement 1B) was calculated using EMAN2 (Tang et al., 2007). Local resolution calculations (Figures 1E and 2B) were performed using blocres (Cardone et al., 2013).

Mtb *RbpA/σ^A^-holo.* The grids were imaged using a 200 keV Talos Arctica (FEI) equipped with a K2 Summit direct electron detector (Gatan). Images were recorded with Serial EM (Mastronarde, 2005) in super-resolution counting mode with a super-resolution pixel size of 0.75 Å and a defocus range of 0.8 μm to 2.4 μm. Data were collected with a dose of 8 electrons/px/s. Images were recorded over a 15 s exposure using 0.3 s subframes (50 total frames) to give a total dose of 53 electrons/Å^2^. Dose-fractionated subframes were 2 × 2 binned (giving a pixel size of 1.5 Å), aligned and summed using Unblur (Grant and Grigorieff, 2015). The contrast transfer function was estimated for each summed image using Gctf (Zhang, 2016). From the summed images, Gautomatch (developed by K. Zhang, MRC Laboratory of Molecular Biology, Cambridge, UK, http://www.mrc-lmb.cam.ac.uk/kzhang/Gautomatch) was used to pick particles with an auto-generated template. Autopicked particles were manually inspected, then subjected to 2D classification in RELION (Scheres, 2012) specifying 100 classes. Poorly populated classes were removed, resulting in a dataset of 289,154 particles. These particles were individually aligned across movie frames and dose-weighted using direct-detector-align_lmbfgs software to generate ‘polished’ particles (Rubinstein and Brubaker, 2015). A subset of the dataset was used to generate an initial model of the complex in cryoSPARC (*ab-initio* reconstruction). ‘Polished’ particles were 3D auto-refined in RELION using this *ab-initio* 3D template (low-pass filtered to 60 Å-resolution). RELION 3D classification into two classes was performed on the particles using the refined map and alignment angles. Among the 3D classes, the best-resolved class, containing 87,657 particles, was 3D auto-refined and post-processed in RELION. The overall resolution of this class was 6.9 Å (before post-processing) and 5.2 Å (after post-processing). Subsequent 3D classification did not improve resolution of this class.

*Fdx/RbpA/σ^A^-holo.* The same procedure as described above for *Mtb* RbpA/σ^A^-holo was used. After RELION 2D classification, poorly populated classes were removed, resulting in a dataset of 63,839 particles. In the end, the best-resolved 3D class, containing 21,115 particles, was 3D auto-refined and post-processed in RELION. The overall resolution of this class was 8.1 Å (before post-processing) and 6.5 Å (after post-processing).

### Model building and refinement

To build initial models of the protein components of the complex, *Msm* RbpA/σ^A^-holo/us-fork structure (PDB ID 5TWI) (Hubin et al., 2017a) was manually fit into the cryo-EM density maps using Chimera (Pettersen et al., 2004) and real-space refined using Phenix (Adams et al., 2010). In the real-space refinement, domains of RNAP were rigid-body refined. For the high-resolution structures, the rigid-body refined models were subsequently refined with secondary structure restraints. A model of Fdx was generated from a crystal structure (Serra et al., 2017), edited in Phenix REEL, and refined into the cryo-EM density. Refined models were inspected and modified in Coot (Emsley and Cowtan, 2004) according to cryo-EM maps, followed by further real-space refinement with PHENIX.

## Additional files

Supplementary file 1. Model statistics.

Supplementary file 2. Superimposition of cryo-EM structures.

Transparent reporting form

### Major datasets

The following datasets were generated:

Boyaci HChen JLilic MDarst SACampbell EA2018Mycobacterium tuberculosis RNAP Holo/RbpA/Fidaxomicinhttp://www.rcsb.org/pdb/search/structidSearch.do?structureId=6C06Publicly available at the RCSB Protein Data Bank (accession no: 6C06)

Boyaci HChen JLilic MDarst SACampbell EA2018Mycobacterium tuberculosis RNAP Holo/RbpA in relaxed statehttp://www.rcsb.org/pdb/search/structidSearch.do?structureId=6C05Publicly available at the RCSB Protein Data Bank (accession no: 6C05)

Boyaci HChen JLilic MDarst SACampbell EA2018Mycobacterium tuberculosis RNAP Holo/RbpA/us-fork DNAhttp://www.rcsb.org/pdb/search/structidSearch.do?structureId=6C04Publicly available at the RCSB Protein Data Bank (accession no: 6C04)

Boyaci HChen JLilic MDarst SACampbell EA2018Mycobacterium tuberculosis Fdx/RNAP Holo/RbpA/us-fork DNAhttp://www.rcsb.org/pdb/search/structidSearch.do?structureId=6BZOPublicly available at the RCSB Protein Data Bank (accession no: 6BZO)

The following previously published datasets were used:

Hubin EADarst SACampbell EA2017Crystal structure of a Mycobacterium smegmatis transcription initiation complex with RbpAhttp://www.rcsb.org/pdb/explore/explore.do?structureId=5tw1Publicly available at the RCSB Protein Data Bank (accession no: 5TW1)

Darst SACampbell EALilic M2017Structure of Mycobacterium smegmatis transcription initiation complex with a full transcription bubblehttps://www.rcsb.org/structure/5vi5Publicly available at the RCSB Protein Data Bank (accession no: 5VI5)

## References

[bib1] Adams PD, Afonine PV, Bunkóczi G, Chen VB, Davis IW, Echols N, Headd JJ, Hung LW, Kapral GJ, Grosse-Kunstleve RW, McCoy AJ, Moriarty NW, Oeffner R, Read RJ, Richardson DC, Richardson JS, Terwilliger TC, Zwart PH (2010). PHENIX: a comprehensive Python-based system for macromolecular structure solution. Acta Crystallographica Section D Biological Crystallography.

[bib2] Bae B, Feklistov A, Lass-Napiorkowska A, Landick R, Darst SA (2015). Structure of a bacterial RNA polymerase holoenzyme open promoter complex. eLife.

[bib3] Belogurov GA, Vassylyeva MN, Sevostyanova A, Appleman JR, Xiang AX, Lira R, Webber SE, Klyuyev S, Nudler E, Artsimovitch I, Vassylyev DG (2009). Transcription inactivation through local refolding of the RNA polymerase structure. Nature.

[bib4] Bernecky C, Herzog F, Baumeister W, Plitzko JM, Cramer P (2016). Structure of transcribing mammalian RNA polymerase II. Nature.

[bib5] Bortoluzzi A, Muskett FW, Waters LC, Addis PW, Rieck B, Munder T, Schleier S, Forti F, Ghisotti D, Carr MD, O'Hare HM (2013). Mycobacterium tuberculosis RNA polymerase-binding protein A (RbpA) and its interactions with sigma factors. Journal of Biological Chemistry.

[bib6] Cardone G, Heymann JB, Steven AC (2013). One number does not fit all: mapping local variations in resolution in cryo-EM reconstructions. Journal of Structural Biology.

[bib7] Cashel M, Hsu LM, Hernandez VJ (2003). Changes in conserved region 3 of Escherichia coli sigma 70 reduce abortive transcription and enhance promoter escape. Journal of Biological Chemistry.

[bib8] Chakraborty A, Wang D, Ebright YW, Korlann Y, Kortkhonjia E, Kim T, Chowdhury S, Wigneshweraraj S, Irschik H, Jansen R, Nixon BT, Knight J, Weiss S, Ebright RH (2012). Opening and closing of the bacterial RNA polymerase clamp. Science.

[bib9] Chakraborty S, Rhee KY (2015). Tuberculosis drug development: history and evolution of the mechanism-based paradigm. Cold Spring Harbor Perspectives in Medicine.

[bib10] Cramer P, Bushnell DA, Kornberg RD (2001). Structural basis of transcription: RNA polymerase II at 2.8 angstrom resolution. Science.

[bib11] Czyz A, Mooney RA, Iaconi A, Landick R (2014). Mycobacterial RNA polymerase requires a U-tract at intrinsic terminators and is aided by NusG at suboptimal terminators. mBio.

[bib12] Davis E, Chen J, Leon K, Darst SA, Campbell EA (2015). Mycobacterial RNA polymerase forms unstable open promoter complexes that are stabilized by CarD. Nucleic Acids Research.

[bib13] Emsley P, Cowtan K (2004). Coot: model-building tools for molecular graphics. Acta Crystallographica Section D Biological Crystallography.

[bib14] Espah Borujeni A, Channarasappa AS, Salis HM (2014). Translation rate is controlled by coupled trade-offs between site accessibility, selective RNA unfolding and sliding at upstream standby sites. Nucleic Acids Research.

[bib15] Feklistov A, Bae B, Hauver J, Lass-Napiorkowska A, Kalesse M, Glaus F, Altmann KH, Heyduk T, Landick R, Darst SA (2017). RNA polymerase motions during promoter melting. Science.

[bib16] Forti F, Mauri V, Dehò G, Ghisotti D (2011). Isolation of conditional expression mutants in Mycobacterium tuberculosis by transposon mutagenesis. Tuberculosis.

[bib17] Gaal T, Ross W, Estrem ST, Nguyen LH, Burgess RR, Gourse RL (2001). Promoter recognition and discrimination by EsigmaS RNA polymerase. Molecular Microbiology.

[bib18] Gnatt AL, Cramer P, Fu J, Bushnell DA, Kornberg RD (2001). Structural basis of transcription: an RNA polymerase II elongation complex at 3.3 A resolution. Science.

[bib19] Grant T, Grigorieff N (2015). Measuring the optimal exposure for single particle cryo-EM using a 2.6 Å reconstruction of rotavirus VP6. eLife.

[bib20] Gualtieri M, Tupin A, Brodolin K, Leonetti JP (2009). Frequency and characterisation of spontaneous lipiarmycin-resistant Enterococcus faecalis mutants selected in vitro. International Journal of Antimicrobial Agents.

[bib21] Gualtieri M, Villain-Guillot P, Latouche J, Leonetti JP, Bastide L (2006). Mutation in the bacillus subtilis RNA polymerase beta' subunit confers resistance to lipiarmycin. Antimicrobial Agents and Chemotherapy.

[bib22] Hoffmann NA, Jakobi AJ, Moreno-Morcillo M, Glatt S, Kosinski J, Hagen WJ, Sachse C, Müller CW (2015). Molecular structures of unbound and transcribing RNA polymerase III. Nature.

[bib23] Hubin EA, Fay A, Xu C, Bean JM, Saecker RM, Glickman MS, Darst SA, Campbell EA (2017a). Structure and function of the mycobacterial transcription initiation complex with the essential regulator RbpA. eLife.

[bib24] Hubin EA, Lilic M, Darst SA, Campbell EA (2017b). Structural insights into the mycobacteria transcription initiation complex from analysis of X-ray crystal structures. Nature Communications.

[bib25] Hubin EA, Tabib-Salazar A, Humphrey LJ, Flack JE, Olinares PD, Darst SA, Campbell EA, Paget MS (2015). Structural, functional, and genetic analyses of the actinobacterial transcription factor RbpA. PNAS.

[bib26] Kadesch TR, Chamberlin MJ (1982). Studies of in vitro transcription by calf thymus RNA polymerase II using a novel duplex DNA template. The Journal of Biological Chemistry.

[bib27] Kang JY, Olinares PD, Chen J, Campbell EA, Mustaev A, Chait BT, Gottesman ME, Darst SA (2017). Structural basis of transcription arrest by coliphage HK022 Nun in an*Escherichia coli*RNA polymerase elongation complex. eLife.

[bib28] Keilty S, Rosenberg M (1987). Constitutive function of a positively regulated promoter reveals new sequences essential for activity. The Journal of Biological Chemistry.

[bib29] Kettenberger H, Armache KJ, Cramer P (2004). Complete RNA polymerase II elongation complex structure and its interactions with NTP and TFIIS. Molecular Cell.

[bib30] Kuehne SA, Dempster AW, Collery MM, Joshi N, Jowett J, Kelly ML, Cave R, Longshaw CM, Minton NP (2017). Characterization of the impact of rpoB mutations on the in vitro and in vivo competitive fitness of Clostridium difficile and susceptibility to fidaxomicin. Journal of Antimicrobial Chemotherapy.

[bib31] Kulbachinskiy A, Mustaev A (2006). Region 3.2 of the sigma subunit contributes to the binding of the 3'-initiating nucleotide in the RNA polymerase active center and facilitates promoter clearance during initiation. Journal of Biological Chemistry.

[bib32] Kurabachew M, Lu SH, Krastel P, Schmitt EK, Suresh BL, Goh A, Knox JE, Ma NL, Jiricek J, Beer D, Cynamon M, Petersen F, Dartois V, Keller T, Dick T, Sambandamurthy VK (2008). Lipiarmycin targets RNA polymerase and has good activity against multidrug-resistant strains of Mycobacterium tuberculosis. Journal of Antimicrobial Chemotherapy.

[bib33] Lane WJ, Darst SA (2010). Molecular evolution of multisubunit RNA polymerases: structural analysis. Journal of Molecular Biology.

[bib34] Mastronarde DN (2005). Automated electron microscope tomography using robust prediction of specimen movements. Journal of Structural Biology.

[bib35] Morichaud Z, Chaloin L, Brodolin K (2016). Regions 1.2 and 3.2 of the RNA polymerase σ subunit promote DNA melting and attenuate action of the antibiotic lipiarmycin. Journal of Molecular Biology.

[bib36] Morin A, Eisenbraun B, Key J, Sanschagrin PC, Timony MA, Ottaviano M, Sliz P (2013). Collaboration gets the most out of software. eLife.

[bib37] Mukhopadhyay J, Das K, Ismail S, Koppstein D, Jang M, Hudson B, Sarafianos S, Tuske S, Patel J, Jansen R, Irschik H, Arnold E, Ebright RH (2008). The RNA polymerase "switch region" is a target for inhibitors. Cell.

[bib38] Murakami KS, Masuda S, Darst SA (2002). Structural basis of transcription initiation: RNA polymerase holoenzyme at 4 A resolution. Science.

[bib39] Neyer S, Kunz M, Geiss C, Hantsche M, Hodirnau VV, Seybert A, Engel C, Scheffer MP, Cramer P, Frangakis AS (2016). Structure of RNA polymerase I transcribing ribosomal DNA genes. Nature.

[bib40] Nicholson WV, White H, Trinick J (2010). An approach to automated acquisition of cryoEM images from lacey carbon grids. Journal of Structural Biology.

[bib41] O'Neill A, Oliva B, Storey C, Hoyle A, Fishwick C, Chopra I (2000). RNA polymerase inhibitors with activity against rifampin-resistant mutants of Staphylococcus aureus. Antimicrobial Agents and Chemotherapy.

[bib42] Pettersen EF, Goddard TD, Huang CC, Couch GS, Greenblatt DM, Meng EC, Ferrin TE (2004). UCSF Chimera--a visualization system for exploratory research and analysis. Journal of Computational Chemistry.

[bib43] Punjani A, Rubinstein JL, Fleet DJ, Brubaker MA (2017). cryoSPARC: algorithms for rapid unsupervised cryo-EM structure determination. Nature Methods.

[bib44] Rosenthal PB, Henderson R (2003). Optimal determination of particle orientation, absolute hand, and contrast loss in single-particle electron cryomicroscopy. Journal of Molecular Biology.

[bib45] Rubinstein JL, Brubaker MA (2015). Alignment of cryo-EM movies of individual particles by optimization of image translations. Journal of Structural Biology.

[bib46] Scheres SH (2012). RELION: implementation of a Bayesian approach to cryo-EM structure determination. Journal of Structural Biology.

[bib47] Schneider CA, Rasband WS, Eliceiri KW (2012). NIH Image to ImageJ: 25 years of image analysis. Nature Methods.

[bib48] Serra S, Malpezzi L, Bedeschi A, Fuganti C, Fonte P (2017). Final demonstration of the co-identity of lipiarmycin A3 and Tiacumicin B (Fidaxomicin) through single crystal X-ray Analysis. Antibiotics.

[bib49] Srivastava A, Talaue M, Liu S, Degen D, Ebright RY, Sineva E, Chakraborty A, Druzhinin SY, Chatterjee S, Mukhopadhyay J, Ebright YW, Zozula A, Shen J, Sengupta S, Niedfeldt RR, Xin C, Kaneko T, Irschik H, Jansen R, Donadio S, Connell N, Ebright RH (2011). New target for inhibition of bacterial RNA polymerase: 'switch region'. Current Opinion in Microbiology.

[bib50] Tabib-Salazar A, Liu B, Doughty P, Lewis RA, Ghosh S, Parsy ML, Simpson PJ, O'Dwyer K, Matthews SJ, Paget MS (2013). The actinobacterial transcription factor RbpA binds to the principal sigma subunit of RNA polymerase. Nucleic Acids Research.

[bib51] Talpaert M, Campagnari F, Clerici L (1975). Lipiarmycin: an antibiotic inhibiting nucleic acid polymerases. Biochemical and Biophysical Research Communications.

[bib52] Tang G, Peng L, Baldwin PR, Mann DS, Jiang W, Rees I, Ludtke SJ (2007). EMAN2: an extensible image processing suite for electron microscopy. Journal of Structural Biology.

[bib53] Tupin A, Gualtieri M, Leonetti JP, Brodolin K (2010). The transcription inhibitor lipiarmycin blocks DNA fitting into the RNA polymerase catalytic site. The EMBO Journal.

[bib54] Vassylyev DG, Vassylyeva MN, Perederina A, Tahirov TH, Artsimovitch I (2007). Structural basis for transcription elongation by bacterial RNA polymerase. Nature.

[bib55] Venugopal AA, Johnson S (2012). Fidaxomicin: a novel macrocyclic antibiotic approved for treatment of Clostridium difficile infection. Clinical Infectious Diseases.

[bib56] Zhang G, Campbell EA, Minakhin L, Richter C, Severinov K, Darst SA (1999). Crystal structure of Thermus aquaticus core RNA polymerase at 3.3 A resolution. Cell.

[bib57] Zhang K (2016). Gctf: Real-time CTF determination and correction. Journal of Structural Biology.

[bib58] Zhang Y, Feng Y, Chatterjee S, Tuske S, Ho MX, Arnold E, Ebright RH (2012). Structural basis of transcription initiation. Science.

[bib59] Zheng SQ, Palovcak E, Armache JP, Verba KA, Cheng Y, Agard DA (2017). MotionCor2: anisotropic correction of beam-induced motion for improved cryo-electron microscopy. Nature Methods.

[bib60] Zumla A, George A, Sharma V, Herbert RH, Oxley A, Oliver M, Baroness Masham of Ilton (2015). The WHO 2014 global tuberculosis report--further to go. The Lancet Global Health.

[bib61] Zuo Y, Steitz TA (2015). Crystal structures of the E. coli transcription initiation complexes with a complete bubble. Molecular Cell.

