# Supplementary material for: Fidaxomicin jams Mycobacterium tuberculosis RNA polymerase motions needed for initiation via RbpA contacts
Source: eLife. 2018 Feb 26;7:e34823. doi: 10.7554/eLife.34823 (PMC5837556; doi:10.7554/eLife.34823)
Supplement: Supplementary file 1. [file elife-34823-supp1.docx]

**Supplementary file 1. Model statistics.**

|  | Fdx/RbpA/σ^A^-holo/us-fork | RbpA/σ^A^-holo/(us-fork)_2_ |
| --- | --- | --- |
| Resolution^a^ | 3.38 Å | 3.27 Å |
| Molprobity score | 2.29 | 2.42 |
| Clashscore (all atoms) | 10.79 | 5.88 |
| Rotamer outliers | 7.13% | 13.48% |
| RMS deviations bonds (Å) | 0.002 | 0.007 |
| RMS deviations angles (°) | 0.491 | 0.837 |
| Ramachandran favored | 89.71% | 92.14% |
| Ramachandran outliers | 0.30% | 0.03% |

^a^ Gold standard FSC 0.143 cutoff criteria (Rosenthal and Henderson, 2003).
