# Supplementary material for: Fidaxomicin jams Mycobacterium tuberculosis RNA polymerase motions needed for initiation via RbpA contacts
Source: eLife. 2018 Feb 26;7:e34823. doi: 10.7554/eLife.34823 (PMC5837556; doi:10.7554/eLife.34823)
Supplement: Supplementary file 2. [file elife-34823-supp2.docx]

**Supplementary file 2. Superimposition of cryo-EM structures.**

| structure | total # of Cα's aligned (structural core)^a^ | rmsd (Å) (structural core alignment) | clamp^b^ rmsd (Å) (461 Cα's) |
| --- | --- | --- | --- |
| RbpA/σ^A^-holo/(us-fork)_2_ (reference) | 1,034 | 0 | 0 |
| RbpA/σ^A^-holo | 900 | 0.386 | 9.01 |
| Fdx/RbpA/σ^A^-holo/us-fork | 898 | 0.384 | 12.4 |
| Fdx/RbpA/σ^A^-holo | 900 | 0.386 | 13.5 |

^a^Structural core module: β residues 30-53, 177-182, 370-380, 445-639, 705-747, 879-1116; β' 414-443, 496-863, 1247-1282; ω.

^b^Clamp module: β residues 1117-1140; β' residues 1-140, 231-413, 1219-1245.
